# Supplementary material for: A cross-sectional study of cardiovascular disease risk clustering at different socio-geographic levels in India
Source: Nat Commun. 2020 Nov 18;11:5891. doi: 10.1038/s41467-020-19647-3 (PMC7674456; doi:10.1038/s41467-020-19647-3)
Supplement: Supplementary file 1 — Supplementary Information [file 41467_2020_19647_MOESM1_ESM.pdf]

Supplementary information

“Clustering of cardiovascular disease risk factors at various socio-geographic levels in India”

Bischops et al.

Supplementary Methods 4
Matching of participants' biomarker data to their socio-demographic information in the Annual Health Survey 4

Supplementary Tables 5
Supplementary Table 1: Sample characteristics of matched and non-matched participants for all AHS states 5

Supplementary Table 2: Sample characteristics of matched and non-matched participants for all Assam 6

Supplementary Table 3: Sample characteristics of matched and non-matched participants for Bihar 6

Supplementary Table 4: Sample characteristics of matched and non-matched participants for Chhattisgarh 7

Supplementary Table 5: Sample characteristics of matched and non-matched participants for Jharkhand 7

Supplementary Table 7: Sample characteristics of matched and non-matched participants for Odisha 8

Supplementary Table 8: Sample characteristics of matched and non-matched participants for Rajasthan 9

Supplementary Table 9: Sample characteristics of matched and non-matched participants for Uttar Pradesh 9

Supplementary Table 10: Sample characteristics of matched and non-matched participants for Uttarakhand 10

Supplementary Table 11: ICCs at the household, community, and district level stratified by wealth quintile and rural versus urban residency 10

Supplementary Table 12: Intracluster correlation coefficients at the household level by state 12

Supplementary Table 13: Intracluster correlation coefficients at the community level by state 14

Supplementary Table 14: Clustering of cardiovascular disease risk factors at the state, district, community, and household level when assuming that all AHS participants were not fasted 16

Supplementary Table 15: Intracluster correlation coefficients at the household level by state when assuming that all AHS participants were not fasted 17

Supplementary Table 16: Intracluster correlation coefficients at the community level by state when assuming that all AHS participants were not fasted 18

Supplementary Table 17. Clustering of BMI, BG and BP as continuous variables at the state, district, community, and household level in India 19

Supplementary Table 18: Intracluster correlation coefficients for BMI, BG and BP as continuous variables at the household level by state 19

Supplementary Table 19: Intracluster correlation coefficients for BMI, BG and BP as continuous variables at the community level by state 20

Supplementary Table 20: Cluster characteristics by state 21

Supplementary Figures 23
Supplementary Figure 1: Intracluster correlation coefficients in relation to CVD risk factor prevalence by state 23

Supplementary Figure 2: Intracluster correlation coefficients in relation to GDP per capita by state 25
Supplementary Figure 3. Intracluster correlation coefficients for BMI, BG and BP as continuous variables at the household and community level by state 27
Supplementary Figure 4: Intracluster correlation coefficients for BMI, BG and BP as continuous variables in relation to household wealth index by district, stratified by residency 28
Supplementary References 30

Supplementary Methods

Matching of participants' biomarker data to their socio-demographic information in the Annual Health Survey

The Annual Health Survey (AHS) made data on participants' physical measurements and their sociodemographic information available in two separate datasets. Because the AHS did not include a unique identifier that would allow us to merge the physical measurements with participant's socio-demographic information, we generated an identifier consisting of state, district, stratum, household identifier (which was unique at the PSU level), and a household member identifier to match these two datasets to each other. 59% of participants in the dataset with physical measurements were successfully matched to their sociodemographic information. Supplementary tables 1 to 10 show that matched and non-matched participants generally had similar characteristics.

Supplementary Tables

Supplementary Table 1: Sample characteristics of matched and non-matched participants for all AHS states

Variable	Matched<br>*n=607,227*	Not matched<br>*n=421,318*
Male (%)	50·7	48·4
Age (mean ± SD)	40·8±15·9	38·9±17·0
Raised blood glucose (%)	7·0	6·3
Plasma glucose in mg/dl (mean ± SD)	108·4±21·7	108·0±21·3
Raised blood pressure (%)	22·3	21·0
Systolic BP (mean ± SD)	123·4±18·9	122·6±19·1
Diastolic BP (mean ± SD)	77·9±12·5	77·3±12·3
Urban (%)	19·4	18·0

Source data are provided as a Source Data file.
Abbr.: BP=blood pressure; SD=standard deviation; mg=milligram; dl= deciliter

Supplementary Table 2: Sample characteristics of matched and non-matched participants for all Assam

Variable	Matched<br>*n=62,882*	Not matched<br>*n=23,626*
Male (%)	51·3	44·5
Age (mean ± SD)	40·3±14·9	37·1±16·1
Raised blood glucose (%)	7·5	7·3
Plasma glucose in mg/dl (mean ± SD)	109·4±21·5	108·5±21·3
Raised blood pressure (%)	24·2	19·7
Systolic BP (mean ± SD)	125·7±17·6	123·3±17·5
Diastolic BP (mean ± SD)	79·5±11·8	77·9±11·5
Urban (%)	16·8	19·8

Source data are provided as a Source Data file.
Abbr.: BP=blood pressure; SD=standard deviation; mg=milligram; dl= deciliter

Supplementary Table 3: Sample characteristics of matched and non-matched participants for Bihar

Variable	Matched<br>*n=71,861*	Not matched<br>*n=80,049*
Male (%)	49·6	53·5
Age (mean ± SD)	40·8±15·9	38·3±16·9
Raised blood glucose (%)	6·9	5·3
Plasma glucose in mg/dl (mean ± SD)	106·7±19·8	104·5±18·6
Raised blood pressure (%)	22·7	18·8
Systolic BP (mean ± SD)	123·7±18·6	120·9±17·5
Diastolic BP (mean ± SD)	77·9±12·8	77·2±11·6
Urban (%)	9·6	8·3

Source data are provided as a Source Data file.
Abbr.: BP=blood pressure; SD=standard deviation; mg=milligram; dl= deciliter

Supplementary Table 4: Sample characteristics of matched and non-matched participants for Chhattisgarh

**Variable**	**Matched** *n=37,579*	**Not matched** *n=19,997*
Male (%)	52·6	48·4
Age (mean ± SD)	39·9±14·9	38·8±16·1
Raised blood glucose (%)	8·8	9·2
Plasma glucose in mg/dl (mean ± SD)	110·0±19·2	110·1±21·0
Raised blood pressure (%)	18·1	18·7
Systolic BP (mean ± SD)	123·1±16·5	123·2±17·2
Diastolic BP (mean ± SD)	77·7±11·9	77·5±12·2
Urban (%)	19·1	22·2

Source data are provided as a Source Data file.
Abbr.: BP=blood pressure; SD=standard deviation; mg=milligram; dl= deciliter

Supplementary Table 5: Sample characteristics of matched and non-matched participants for Jharkhand

**Variable**	**Matched** *n=35,721*	**Not matched** *n=18,875*
Male (%)	44·8	43·6
Age (mean ± SD)	40·6±15·5	39·1±16·8
Raised blood glucose (%)	6·8	5·4
Plasma glucose in mg/dl (mean ± SD)	105·4±24·8	103·9±21·7
Raised blood pressure (%)	25·0	24·3
Systolic BP (mean ± SD)	123·0±20·0	122·7±19·9
Diastolic BP (mean ± SD)	77·8±13·3	77·5±13·2
Urban (%)	17·6	22·2

Source data are provided as a Source Data file.
Abbr.: BP=blood pressure; SD=standard deviation; mg=milligram; dl= deciliter

Supplementary Table 6: Sample characteristics of matched and non-matched participants for Madhya Pradesh

**Variable**	**Matched**<br><br>*n=101,896*	**Not matched**<br><br>*n=63,056*
Male (%)	54·4	51·1
Age (mean ± SD)	40·0±15·8	38·9±16·8
Raised blood glucose (%)	6·2	5·4
Plasma glucose in mg/dl (mean ± SD	107·3±20·0	107·3±19·0
Raised blood pressure (%)	21·7	21·2
Systolic BP (mean ± SD)	124·6±18·1	124·3±17·8
Diastolic BP (mean ± SD)	79·8±11·4	79·6±11·1
Urban (%)	32·4	28·9

Source data are provided as a Source Data file.
Abbr.: BP=blood pressure; SD=standard deviation; mg=milligram; dl= deciliter

Supplementary Table 7: Sample characteristics of matched and non-matched participants for Odisha

**Variable**	**Matched**<br><br>*n=92,000*	**Not matched**<br><br>*n=21,570*
Male (%)	49·2	43·8
Age (mean ± SD)	42·2±16·0	39·2±17·5
Raised blood glucose (%)	7·0	6·4
Plasma glucose in mg/dl (mean ± SD)	107·6±24·0	106·3±23·2
Raised blood pressure (%)	20·2	18·6
Systolic BP (mean ± SD)	120·7±19·8	119·6±19·8
Diastolic BP (mean ± SD)	75·4±13·2	74·8±13·1
Urban (%)	14·3	13·2

Source data are provided as a Source Data file.
Abbr.: BP=blood pressure; SD=standard deviation; mg=milligram; dl= deciliter

Supplementary Table 8: Sample characteristics of matched and non-matched participants for Rajasthan

**Variable**	**Matched**<br><br>*n=81,931*	**Not matched**<br><br>*n=25,974*
Male (%)	49·6	44·6
Age (mean ± SD)	41·0±16·1	37·9±17·5
Raised blood glucose (%)	6·9	6·2
Plasma glucose in mg/dl (mean ± SD)	109·7±20·4	109·0±19·7
Raised blood pressure (%)	22·8	20·9
Systolic BP (mean ± SD)	122·9±18·5	121·9±18·3
Diastolic BP (mean ± SD)	78·7±11·9	78·2±11·8
Urban (%)	17·7	17·1

Source data are provided as a Source Data file.
Abbr.: BP=blood pressure; SD=standard deviation; mg=milligram; dl= deciliter

Supplementary Table 9: Sample characteristics of matched and non-matched participants for Uttar Pradesh

**Variable**	**Matched**<br><br>*n=103,384*	**Not matched**<br><br>*n=148,213*
Male (%)	51·6	47·9
Age (mean ± SD)	40·4±16·3	39·4±17·2
Raised blood glucose (%)	6·9	6·2
Plasma glucose in mg/dl (mean ± SD)	110·4±22·0	110·2±22·3
Raised blood pressure (%)	21·8	22·0
Systolic BP (mean ± SD)	123·1±20·0	122·9±20·6
Diastolic BP (mean ± SD)	76·2±13·0	76·1±13·0
Urban (%)	21·3	17·5

Source data are provided as a Source Data file.
Abbr.: BP=blood pressure; SD=standard deviation; mg=milligram; dl= deciliter

Supplementary Table 10: Sample characteristics of matched and non-matched participants for Uttarakhand

**Variable**	**Matched**<br>*n=19,973*	**Not matched**<br>*n=19,958*
Male (%)	47·0	42·6
Age (mean ± SD)	42·7±16·6	40·1±17·3
Raised blood glucose (%)	8·0	7·3
Plasma glucose in mg/dl (mean ± SD)	109·6±25·2	111·3±25·9
Raised blood pressure (%)	32·1	25·9
Systolic BP (mean ± SD)	127·2±19·6	124·0±20·1
Diastolic BP (mean ± SD)	81·5±11·8	79·2±12·3
Urban (%)	22·0	21·3

Source data are provided as a Source Data file.
Abbr.: BP=blood pressure; SD=standard deviation; mg=milligram; dl= deciliter

Supplementary Table 11: ICCs at the household, community, and district level stratified by wealth quintile and rural versus urban residency

	Wealth quintile	Household		Community		District	
		Rural (95% CI)	Urban (95% CI)	Rural (95% CI)	Urban (95% CI)	Rural (95% CI)	Urban (95% CI)
Raised blood glucose	1	0.148 (0.141 to 0.156)	0.132 (0.124 to 0.142)	0.044 (0.040 to 0.049)	0.076 (0.069 to 0.083)	0.014 (0.010 to 0.018)	0.028 (0.022 to 0.036)
	2	0.151 (0.144 to 0.157)	0.146 (0.137 to 0.155)	0.062 (0.057 to 0.067)	0.065 (0.059 to 0.071)	0.016 (0.012 to 0.020)	0.032 (0.024 to 0.042)
	3	0.151 (0.145 to 0.157)	0.135 (0.127 to 0.144)	0.083 (0.078 to 0.088)	0.075 (0.069 to 0.082)	0.024 (0.018 to 0.031)	0.036 (0.026 to 0.047)
	4	0.145 (0.140 to 0.152)	0.142 (0.134 to 0.150)	0.080 (0.076 to 0.084)	0.080 (0.073 to 0.087)	0.036 (0.027 to 0.046)	0.034 (0.025 to 0.044)
	5	0.136 (0.130 to 0.142)	0.127 (0.120 to 0.136)	0.066 (0.061 to 0.069)	0.060 (0.055 to 0.066)	0.034 (0.026 to 0.043)	0.011 (0.007 to 0.017)
Raised blood pressure	1	0.111 (0.103 to 0.117)	0.101 (0.092 to 0.110)	0.067 (0.061 to 0.074)	0.056 (0.050 to 0.061)	0.027 (0.020 to 0.034)	0.044 (0.036 to 0.056)
	2	0.114 (0.108 to 0.121)	0.096 (0.088 to 0.105)	0.064 (0.059 to 0.070)	0.042 (0.038 to 0.047)	0.025 (0.019 to 0.031)	0.017 (0.012 to 0.024)
	3	0.097 (0.090 to 0.102)	0.085 (0.076 to 0.093)	0.053 (0.049 to 0.057)	0.046 (0.041 to 0.050)	0.031 (0.023 to 0.040)	0.028 (0.020 to 0.038)

	4	0.101 (0.095 to 0.107)	0.089 (0.080 to 0.097)	0.051 (0.048 to 0.055)	0.055 (0.050 to 0.060)	0.016 (0.012 to 0.021)	0.028 (0.019 to 0.035)
	5	0.097 (0.091 to 0.102)	0.080 (0.072 to 0.088)	0.063 (0.059 to 0.067)	0.061 (0.055 to 0.067)	0.048 (0.037 to 0.062)	0.047 (0.034 to 0.065)
Current smoking	1	0.043 (0.036 to 0.049)	0.065 (0.057 to 0.075)	0.134 (0.124 to 0.144)	0.116 (0.106 to 0.126)	0.040 (0.031 to 0.051)	0.064 (0.052 to 0.076)
	2	0.072 (0.065 to 0.078)	0.082 (0.074 to 0.091)	0.151 (0.141 to 0.162)	0.096 (0.087 to 0.103)	0.051 (0.040 to 0.064)	0.034 (0.026 to 0.043)
	3	0.091 (0.085 to 0.097)	0.106 (0.097 to 0.114)	0.142 (0.133 to 0.150)	0.106 (0.098 to 0.114)	0.069 (0.052 to 0.089)	0.060 (0.041 to 0.078)
	4	0.096 (0.090 to 0.102)	0.096 (0.088 to 0.104)	0.125 (0.119 to 0.131)	0.117 (0.108 to 0.128)	0.075 (0.056 to 0.095)	0.121 (0.089 to 0.153)
	5	0.127 (0.120 to 0.132)	0.105 (0.098 to 0.113)	0.137 (0.130 to 0.144)	0.130 (0.119 to 0.140)	0.097 (0.074 to 0.127)	0.041 (0.028 to 0.058)
Overweight	1	0.171 (0.164 to 0.177)	0.185 (0.176 to 0.194)	0.070 (0.064 to 0.076)	0.075 (0.068 to 0.083)	0.042 (0.031 to 0.055)	0.078 (0.064 to 0.094)
	2	0.156 (0.150 to 0.163)	0.183 (0.175 to 0.191)	0.075 (0.069 to 0.081)	0.063 (0.057 to 0.069)	0.023 (0.018 to 0.029)	0.054 (0.042 to 0.071)
	3	0.161 (0.154 to 0.168)	0.171 (0.162 to 0.178)	0.054 (0.050 to 0.058)	0.071 (0.065 to 0.077)	0.023 (0.017 to 0.030)	0.047 (0.033 to 0.063)
	4	0.169 (0.163 to 0.175)	0.187 (0.179 to 0.196)	0.058 (0.054 to 0.062)	0.071 (0.065 to 0.077)	0.022 (0.017 to 0.028)	0.063 (0.047 to 0.083)
	5	0.174 (0.167 to 0.180)	0.189 (0.181 to 0.197)	0.069 (0.065 to 0.074)	0.076 (0.070 to 0.083)	0.044 (0.033 to 0.054)	0.034 (0.022 to 0.046)
Obesity	1	0.084 (0.078 to 0.090)	0.132 (0.122 to 0.141)	0.019 (0.017 to 0.021)	0.072 (0.065 to 0.079)	0.011 (0.008 to 0.014)	0.050 (0.040 to 0.060)
	2	0.087 (0.080 to 0.094)	0.154 (0.145 to 0.164)	0.029 (0.026 to 0.032)	0.050 (0.045 to 0.055)	0.013 (0.010 to 0.016)	0.035 (0.025 to 0.045)
	3	0.102 (0.097 to 0.108)	0.133 (0.125 to 0.141)	0.030 (0.027 to 0.033)	0.056 (0.051 to 0.063)	0.011 (0.008 to 0.014)	0.032 (0.023 to 0.043)
	4	0.113 (0.107 to 0.120)	0.155 (0.146 to 0.163)	0.039 (0.036 to 0.042)	0.059 (0.054 to 0.065)	0.012 (0.009 to 0.016)	0.043 (0.030 to 0.056)
	5	0.141 (0.136 to 0.147)	0.182 (0.174 to 0.191)	0.046 (0.043 to 0.050)	0.074 (0.068 to 0.081)	0.029 (0.022 to 0.038)	0.030 (0.020 to 0.042)

Source data are provided as a Source Data file.

Supplementary Table 12: Intracluster correlation coefficients at the household level by state

State	Intracluster correlation coefficient				
	Raised blood glucose (95% CI)	Raised blood pressure (95% CI)	Smoking (95% CI)	Overweight (95% CI)	Obesity (95% CI)
Andaman and Nicobar Islands	0.204 (0.167 to 0.242)	0.16 (0.121 to 0.198)	0.09 (0.054 to 0.126)	0.207 (0.171 to 0.246)	0.145 (0.111 to 0.182)
Andhra Pradesh	0.13 (0.116 to 0.142)	0.095 (0.082 to 0.109)	0.102 (0.089 to 0.116)	0.216 (0.201 to 0.229)	0.181 (0.167 to 0.195)
Arunachal Pradesh	0.201 (0.188 to 0.215)	0.135 (0.121 to 0.148)	0.086 (0.074 to 0.098)	0.189 (0.178 to 0.202)	0.13 (0.116 to 0.145)
Assam	0.088 (0.077 to 0.101)	0.066 (0.054 to 0.077)	0 (0.000 to 0.011)	0.245 (0.233 to 0.257)	0.138 (0.124 to 0.149)
Bihar	0.104 (0.091 to 0.117)	0.099 (0.087 to 0.112)	0.054 (0.040 to 0.066)	0.113 (0.100 to 0.125)	0.047 (0.035 to 0.059)
Chandigarh	0.122 (0.082 to 0.164)	0 (0.000 to 0.000)	0.043 (0.006 to 0.078)	0.171 (0.131 to 0.209)	0.067 (0.031 to 0.108)
Chhattisgarh	0.136 (0.122 to 0.152)	0.094 (0.080 to 0.108)	0.017 (0.005 to 0.030)	0.206 (0.192 to 0.220)	0.146 (0.131 to 0.161)
Daman and Diu	0.287 (0.227 to 0.347)	0.209 (0.149 to 0.265)	0.011 (0.000 to 0.062)	0.192 (0.130 to 0.260)	0.225 (0.164 to 0.283)
Delhi	0.116 (0.097 to 0.135)	0.2 (0.179 to 0.219)	0.113 (0.095 to 0.131)	0.144 (0.123 to 0.164)	0.135 (0.117 to 0.153)
Goa	0.162 (0.126 to 0.196)	0.169 (0.134 to 0.203)	0.186 (0.151 to 0.222)	0.206 (0.170 to 0.242)	0.13 (0.098 to 0.163)
Haryana	0.091 (0.082 to 0.101)	0.071 (0.061 to 0.081)	0.061 (0.052 to 0.071)	0.196 (0.187 to 0.207)	0.16 (0.150 to 0.171)
Himachal Pradesh	0.117 (0.094 to 0.138)	0.087 (0.065 to 0.106)	0.013 (0.000 to 0.034)	0.197 (0.172 to 0.221)	0.103 (0.083 to 0.125)
Jharkhand	0.067 (0.051 to 0.083)	0.093 (0.077 to 0.110)	0.098 (0.081 to 0.113)	0.238 (0.222 to 0.255)	0.121 (0.104 to 0.139)
Karnataka	0.135 (0.128 to 0.141)	0.06 (0.054 to 0.067)	0.034 (0.027 to 0.041)	0.221 (0.214 to 0.229)	0.173 (0.165 to 0.180)
Kerala	0.146 (0.128 to 0.163)	0.222 (0.207 to 0.240)	0 (0.000 to 0.016)	0.19 (0.172 to 0.209)	0.182 (0.163 to 0.197)
Madhya Pradesh	0.088 (0.078 to 0.097)	0.06 (0.050 to 0.069)	0 (0.000 to 0.009)	0.178 (0.168 to 0.188)	0.098 (0.087 to 0.108)
Maharashtra	0.179 (0.172 to 0.187)	0.134 (0.125 to 0.141)	0.128 (0.120 to 0.136)	0.202 (0.194 to 0.211)	0.163 (0.156 to 0.171)
Manipur	0.133 (0.116 to 0.151)	0.08 (0.064 to 0.094)	0.029 (0.014 to 0.044)	0.141 (0.125 to 0.159)	0.104 (0.089 to 0.120)
Meghalaya	0.253 (0.226 to 0.275)	0.139 (0.116 to 0.164)	0 (0.000 to 0.023)	0.169 (0.145 to 0.194)	0.127 (0.101 to 0.150)
Mizoram	0.099 (0.084 to 0.115)	0.061 (0.046 to 0.076)	0.046 (0.032 to 0.059)	0.17 (0.153 to 0.186)	0.103 (0.088 to 0.118)
Nagaland	0.125 (0.100 to 0.150)	0.112 (0.090 to 0.134)	0 (0.000 to 0.000)	0.193 (0.167 to 0.216)	0.098 (0.074 to 0.120)
Odisha	0.086 (0.077 to 0.095)	0.104 (0.094 to 0.114)	0.018 (0.009 to 0.027)	0.265 (0.255 to 0.274)	0.156 (0.147 to 0.166)
Puducherry	0.114 (0.095 to 0.134)	0.057 (0.038 to 0.078)	0.042 (0.022 to 0.061)	0.16 (0.137 to 0.183)	0.136 (0.114 to 0.158)
Punjab	0.108 (0.102 to 0.115)	0.051 (0.044 to 0.058)	0.066 (0.060 to 0.073)	0.165 (0.157 to 0.172)	0.147 (0.140 to 0.156)
Rajasthan	0.11 (0.099 to 0.122)	0.074 (0.064 to 0.086)	0.038 (0.028 to 0.050)	0.198 (0.186 to 0.209)	0.126 (0.114 to 0.138)
Sikkim	0.13 (0.104 to 0.154)	0.084 (0.060 to 0.109)	0.095 (0.071 to 0.121)	0.178 (0.153 to 0.205)	0.147 (0.123 to 0.173)
Tamil Nadu	0.12 (0.113 to 0.128)	0.069 (0.062 to 0.076)	0.031 (0.023 to 0.038)	0.196 (0.188 to 0.204)	0.152 (0.144 to 0.161)

Telangana	0.113 (0.096 to 0.132)	0.11 (0.092 to 0.125)	0.094 (0.079 to 0.110)	0.222 (0.207 to 0.238)	0.18 (0.164 to 0.199)
Tripura	0.104 (0.075 to 0.134)	0.068 (0.037 to 0.095)	0.092 (0.065 to 0.122)	0.137 (0.108 to 0.166)	0.096 (0.065 to 0.124)
Uttar Pradesh	0.114 (0.102 to 0.125)	0.133 (0.123 to 0.143)	0 (0.000 to 0.009)	0.184 (0.174 to 0.193)	0.121 (0.110 to 0.131)
Uttarakhand	0.131 (0.108 to 0.156)	0.103 (0.080 to 0.124)	0 (0.000 to 0.021)	0.286 (0.262 to 0.310)	0.14 (0.114 to 0.167)
West Bengal	0.15 (0.139 to 0.163)	0.08 (0.070 to 0.091)	0.012 (0.001 to 0.023)	0.235 (0.222 to 0.248)	0.157 (0.146 to 0.168)

Supplementary Table 13: Intracluster correlation coefficients at the community level by state

State	Intracluster correlation coefficient at the community level				
	Raised blood glucose (95% CI)	Raised blood pressure (95% CI)	Smoking (95% CI)	Overweight (95% CI)	Obesity (95% CI)
Andaman and Nicobar Islands	0.086 (0.062 to 0.114)	0.065 (0.043 to 0.087)	0.055 (0.036 to 0.076)	0.063 (0.041 to 0.084)	0.049 (0.032 to 0.069)
Andhra Pradesh	0.044 (0.037 to 0.051)	0.032 (0.027 to 0.037)	0.077 (0.067 to 0.086)	0.093 (0.082 to 0.103)	0.078 (0.069 to 0.088)
Arunachal Pradesh	0.082 (0.073 to 0.093)	0.064 (0.056 to 0.074)	0.090 (0.078 to 0.101)	0.088 (0.077 to 0.099)	0.056 (0.048 to 0.064)
Assam	0.036 (0.029 to 0.043)	0.056 (0.045 to 0.067)	0.067 (0.055 to 0.079)	0.143 (0.122 to 0.165)	0.081 (0.068 to 0.096)
Bihar	0.062 (0.052 to 0.072)	0.060 (0.051 to 0.071)	0.045 (0.038 to 0.053)	0.069 (0.058 to 0.080)	0.029 (0.024 to 0.035)
Chandigarh	0.030 (0.015 to 0.049)	0.022 (0.008 to 0.035)	0.070 (0.042 to 0.102)	0.103 (0.068 to 0.146)	0.031 (0.016 to 0.049)
Chhattisgarh	0.048 (0.037 to 0.060)	0.038 (0.029 to 0.047)	0.049 (0.037 to 0.060)	0.102 (0.082 to 0.123)	0.059 (0.047 to 0.074)
Daman and Diu	0.098 (0.056 to 0.145)	0.086 (0.047 to 0.132)	0.013 (0.000 to 0.030)	0.034 (0.011 to 0.058)	0.064 (0.034 to 0.102)
Delhi	0.035 (0.027 to 0.042)	0.084 (0.070 to 0.099)	0.160 (0.137 to 0.182)	0.066 (0.055 to 0.078)	0.051 (0.041 to 0.061)
Goa	0.060 (0.040 to 0.082)	0.070 (0.049 to 0.096)	0.056 (0.038 to 0.080)	0.034 (0.020 to 0.049)	0.037 (0.023 to 0.055)
Haryana	0.036 (0.032 to 0.041)	0.038 (0.033 to 0.042)	0.077 (0.069 to 0.084)	0.079 (0.071 to 0.086)	0.061 (0.054 to 0.067)
Himachal Pradesh	0.029 (0.021 to 0.038)	0.036 (0.028 to 0.045)	0.071 (0.059 to 0.084)	0.122 (0.103 to 0.140)	0.044 (0.034 to 0.055)
Jharkhand	0.028 (0.021 to 0.036)	0.041 (0.032 to 0.050)	0.059 (0.046 to 0.071)	0.130 (0.107 to 0.154)	0.080 (0.065 to 0.098)
Karnataka	0.061 (0.056 to 0.066)	0.025 (0.022 to 0.028)	0.049 (0.045 to 0.054)	0.103 (0.095 to 0.110)	0.077 (0.071 to 0.083)
Kerala	0.098 (0.086 to 0.111)	0.169 (0.150 to 0.187)	0.144 (0.129 to 0.161)	0.105 (0.091 to 0.120)	0.097 (0.084 to 0.111)
Madhya Pradesh	0.043 (0.037 to 0.050)	0.041 (0.035 to 0.047)	0.057 (0.050 to 0.065)	0.081 (0.070 to 0.091)	0.043 (0.037 to 0.050)
Maharashtra	0.088 (0.082 to 0.094)	0.066 (0.061 to 0.071)	0.079 (0.073 to 0.085)	0.101 (0.094 to 0.108)	0.068 (0.063 to 0.074)
Manipur	0.042 (0.034 to 0.050)	0.049 (0.039 to 0.058)	0.085 (0.071 to 0.101)	0.066 (0.055 to 0.079)	0.039 (0.031 to 0.047)
Meghalaya	0.076 (0.062 to 0.093)	0.073 (0.058 to 0.089)	0.050 (0.038 to 0.062)	0.073 (0.058 to 0.089)	0.044 (0.031 to 0.055)
Mizoram	0.028 (0.022 to 0.035)	0.027 (0.021 to 0.033)	0.038 (0.031 to 0.046)	0.057 (0.047 to 0.068)	0.032 (0.025 to 0.040)
Nagaland	0.039 (0.029 to 0.049)	0.081 (0.066 to 0.097)	0.076 (0.060 to 0.091)	0.093 (0.076 to 0.110)	0.041 (0.032 to 0.051)
Odisha	0.019 (0.016 to 0.023)	0.054 (0.046 to 0.063)	0.052 (0.045 to 0.060)	0.115 (0.100 to 0.131)	0.069 (0.058 to 0.079)
Puducherry	0.027 (0.018 to 0.035)	0.016 (0.010 to 0.022)	0.028 (0.019 to 0.036)	0.052 (0.039 to 0.067)	0.037 (0.027 to 0.048)
Punjab	0.038 (0.035 to 0.042)	0.034 (0.030 to 0.037)	0.056 (0.051 to 0.062)	0.054 (0.049 to 0.059)	0.044 (0.040 to 0.048)
Rajasthan	0.054 (0.046 to 0.064)	0.047 (0.040 to 0.055)	0.092 (0.080 to 0.105)	0.119 (0.103 to 0.135)	0.105 (0.091 to 0.122)
Sikkim	0.027 (0.018 to 0.036)	0.029 (0.019 to 0.039)	0.030 (0.019 to 0.042)	0.042 (0.029 to 0.053)	0.036 (0.025 to 0.048)
Tamil Nadu	0.044 (0.040 to 0.048)	0.023 (0.020 to 0.026)	0.053 (0.049 to 0.058)	0.073 (0.068 to 0.079)	0.055 (0.051 to 0.060)

Telangana	0.049 (0.041 to 0.058)	0.048 (0.038 to 0.056)	0.113 (0.099 to 0.128)	0.097 (0.085 to 0.110)	0.073 (0.062 to 0.084)
Tripura	0.050 (0.033 to 0.069)	0.030 (0.018 to 0.044)	0.093 (0.067 to 0.120)	0.034 (0.021 to 0.050)	0.023 (0.012 to 0.035)
Uttar Pradesh	0.042 (0.035 to 0.048)	0.078 (0.069 to 0.089)	0.055 (0.048 to 0.063)	0.126 (0.112 to 0.140)	0.089 (0.078 to 0.101)
Uttarakhand	0.056 (0.040 to 0.072)	0.081 (0.059 to 0.107)	0.074 (0.055 to 0.094)	0.170 (0.135 to 0.206)	0.080 (0.059 to 0.103)
West Bengal	0.066 (0.059 to 0.074)	0.049 (0.043 to 0.055)	0.055 (0.049 to 0.063)	0.125 (0.112 to 0.136)	0.075 (0.067 to 0.083)

Source data are provided as a Source Data file.

Supplementary Table 14: Clustering of cardiovascular disease risk factors at the state, district, community, and household level when assuming that all AHS participants were not fasted

Risk factor	Intracluster Correlation Coefficient			
	State (95% CI)	*District (95% CI)*	*Community (95% CI)*	*Household (95% CI)*
Raised BG	0.054 (0.032 to 0.078)	0.056 (0.050 to 0.062)	0.116 (0.113 to 0.118)	0.156 (0.153 to 0.158)
Raised BP	0.023 (0.012 to 0.036)	0.034 (0.030 to 0.038)	0.065 (0.063 to 0.067)	0.104 (0.102 to 0.107)
Current smoker	0.090 (0.048 to 0.140)	0.063 (0.056 to 0.070)	0.131 (0.128 to 0.134)	0.095 (0.093 to 0.097)
Overweight	0.045 (0.023 to 0.072)	0.073 (0.065 to 0.081)	0.134 (0.132 to 0.137)	0.236 (0.234 to 0.239)
Obesity	0.029 (0.015 to 0.046)	0.039 (0.034 to 0.044)	0.099 (0.096 to 0.101)	0.165 (0.163 to 0.167)

Source data are provided as a Source Data file.

Abbr.: CI=Confidence interval; BG=blood glucose; BP=blood pressure

Supplementary Table 15: Intracluster correlation coefficients at the household level by state when assuming that all AHS participants were not fasted

State	Intracluster correlation coefficient
	Raised blood glucose (95% CI)
Andaman and Nicobar Islands	0.204 (0.168 to 0.247)
Andhra Pradesh	0.130 (0.116 to 0.142)
Arunachal Pradesh	0.201 (0.187 to 0.213)
Assam	0.056 (0.043 to 0.068)
Bihar	0.048 (0.034 to 0.060)
Chandigarh	0.122 (0.084 to 0.166)
Chhattisgarh	0.006 (0.000 to 0.019)
Daman and Diu	0.287 (0.220 to 0.342)
Delhi	0.116 (0.097 to 0.134)
Goa	0.162 (0.126 to 0.197)
Haryana	0.091 (0.081 to 0.100)
Himachal Pradesh	0.117 (0.094 to 0.141)
Jharkhand	0.033 (0.017 to 0.048)
Karnataka	0.135 (0.127 to 0.142)
Kerala	0.146 (0.130 to 0.163)
Madhya Pradesh	0.042 (0.033 to 0.051)
Maharashtra	0.179 (0.171 to 0.187)
Manipur	0.133 (0.117 to 0.151)
Meghalaya	0.253 (0.230 to 0.277)
Mizoram	0.099 (0.085 to 0.115)
Nagaland	0.125 (0.101 to 0.146)
Odisha	0.021 (0.013 to 0.031)
Puducherry	0.114 (0.093 to 0.133)
Punjab	0.108 (0.100 to 0.115)
Rajasthan	0.029 (0.018 to 0.039)
Sikkim	0.130 (0.105 to 0.155)
Tamil Nadu	0.120 (0.113 to 0.129)
Telangana	0.113 (0.096 to 0.129)
Tripura	0.104 (0.078 to 0.134)
Uttar Pradesh	0.040 (0.032 to 0.050)
Uttarakhand	0.044 (0.022 to 0.066)
West Bengal	0.150 (0.138 to 0.162)

Source data are provided as a Source Data file.

Supplementary Table 16: Intracluster correlation coefficients at the community level by state when assuming that all AHS participants were not fasted

State	Intracluster correlation coefficient
	Raised blood glucose (95% CI)
Andaman and Nicobar Islands	0.086 (0.063 to 0.114)
Andhra Pradesh	0.044 (0.037 to 0.050)
Arunachal Pradesh	0.082 (0.072 to 0.094)
Assam	0.010 (0.007 to 0.013)
Bihar	0.011 (0.008 to 0.014)
Chandigarh	0.030 (0.013 to 0.047)
Chhattisgarh	0.005 (0.003 to 0.007)
Daman and Diu	0.098 (0.054 to 0.151)
Delhi	0.035 (0.026 to 0.042)
Goa	0.060 (0.041 to 0.080)
Haryana	0.036 (0.032 to 0.041)
Himachal Pradesh	0.029 (0.021 to 0.038)
Jharkhand	0.011 (0.007 to 0.015)
Karnataka	0.061 (0.056 to 0.066)
Kerala	0.098 (0.084 to 0.112)
Madhya Pradesh	0.011 (0.009 to 0.013)
Maharashtra	0.088 (0.082 to 0.094)
Manipur	0.042 (0.034 to 0.050)
Meghalaya	0.076 (0.060 to 0.093)
Mizoram	0.028 (0.022 to 0.034)
Nagaland	0.039 (0.029 to 0.050)
Odisha	0.006 (0.004 to 0.007)
Puducherry	0.027 (0.019 to 0.036)
Punjab	0.038 (0.034 to 0.042)
Rajasthan	0.008 (0.005 to 0.010)
Sikkim	0.027 (0.018 to 0.036)
Tamil Nadu	0.044 (0.040 to 0.048)
Telangana	0.049 (0.040 to 0.059)
Tripura	0.050 (0.033 to 0.067)
Uttar Pradesh	0.010 (0.008 to 0.012)
Uttarakhand	0.016 (0.010 to 0.024)
West Bengal	0.066 (0.059 to 0.074)

Source data are provided as a Source Data file.

Supplementary Table 17. Clustering of BMI, BG and BP as continuous variables at the state, district, community, and household level in India

Risk factor	Intracluster Correlation Coefficient			
	State (95% CI)	*District (95% CI)*	*Community (95% CI)*	*Household (95% CI)*
BG	0.083 (0.047 to 0.120)	0.102 (0.091 to 0.112)	0.129 (0.127 to 0.132)	0.184 (0.182 to 0.186)
BP	0.023 (0.013 to 0.037)	0.046 (0.041 to 0.051)	0.076 (0.074 to 0.078)	0.138 (0.136 to 0.141)
BMI	0.055 (0.031 to 0.082)	0.072 (0.064 to 0.080)	0.153 (0.150 to 0.156)	0.278 (0.276 to 0.281)

Source data are provided as a Source Data file.
Abbr.: CI=Confidence interval; BG=blood glucose; BP=blood pressure

Supplementary Table 18: Intracluster correlation coefficients for BMI, BG and BP as continuous variables at the household level by state

State	Intracluster Correlation Coefficient		
	Blood glucose (95% CI)	Blood pressure (95% CI)	BMI (95% CI)
Andaman and Nicobar Islands	0.109 (0.071 to 0.144)	0.191 (0.154 to 0.232)	0.273 (0.236 to 0.309)
Andhra Pradesh	0.132 (0.119 to 0.145)	0.113 (0.101 to 0.127)	0.271 (0.259 to 0.286)
Arunachal Pradesh	0.183 (0.168 to 0.197)	0.142 (0.129 to 0.156)	0.220 (0.207 to 0.233)
Assam	0.121 (0.109 to 0.133)	0.102 (0.090 to 0.113)	0.331 (0.317 to 0.342)
Bihar	0.220 (0.207 to 0.233)	0.138 (0.125 to 0.151)	0.181 (0.168 to 0.193)
Chandigarh	0.135 (0.096 to 0.176)	0.027 (0.000 to 0.063)	0.152 (0.113 to 0.193)
Chhattisgarh	0.127 (0.113 to 0.142)	0.116 (0.101 to 0.130)	0.247 (0.232 to 0.261)
Daman and Diu	0.173 (0.111 to 0.237)	0.267 (0.207 to 0.330)	0.250 (0.181 to 0.311)
Delhi	0.065 (0.049 to 0.082)	0.114 (0.094 to 0.132)	0.180 (0.160 to 0.198)
Goa	0.188 (0.154 to 0.224)	0.171 (0.136 to 0.207)	0.191 (0.154 to 0.225)
Haryana	0.107 (0.097 to 0.116)	0.099 (0.090 to 0.109)	0.244 (0.233 to 0.253)
Himachal Pradesh	0.109 (0.085 to 0.129)	0.151 (0.130 to 0.174)	0.194 (0.173 to 0.217)
Jharkhand	0.080 (0.064 to 0.096)	0.125 (0.108 to 0.141)	0.269 (0.251 to 0.286)
Karnataka	0.160 (0.153 to 0.168)	0.078 (0.071 to 0.084)	0.286 (0.279 to 0.294)
Kerala	0.103 (0.086 to 0.121)	0.194 (0.179 to 0.211)	0.260 (0.243 to 0.275)
Madhya Pradesh	0.159 (0.150 to 0.167)	0.169 (0.159 to 0.178)	0.248 (0.237 to 0.257)
Maharashtra	0.135 (0.127 to 0.142)	0.120 (0.113 to 0.127)	0.242 (0.234 to 0.250)
Manipur	0.188 (0.172 to 0.204)	0.099 (0.084 to 0.115)	0.212 (0.195 to 0.229)
Meghalaya	0.157 (0.131 to 0.183)	0.156 (0.131 to 0.181)	0.202 (0.179 to 0.227)
Mizoram	0.239 (0.223 to 0.255)	0.095 (0.079 to 0.109)	0.223 (0.208 to 0.239)
Nagaland	0.251 (0.227 to 0.273)	0.145 (0.122 to 0.169)	0.213 (0.189 to 0.238)
Odisha	0.115 (0.106 to 0.125)	0.182 (0.172 to 0.191)	0.343 (0.334 to 0.354)
Puducherry	0.145 (0.123 to 0.168)	0.078 (0.057 to 0.098)	0.202 (0.178 to 0.221)
Punjab	0.079 (0.072 to 0.086)	0.076 (0.070 to 0.084)	0.198 (0.191 to 0.205)
Rajasthan	0.166 (0.154 to 0.178)	0.131 (0.120 to 0.144)	0.265 (0.254 to 0.276)
Sikkim	0.141 (0.114 to 0.166)	0.093 (0.069 to 0.115)	0.208 (0.181 to 0.230)
Tamil Nadu	0.105 (0.097 to 0.113)	0.103 (0.096 to 0.111)	0.244 (0.235 to 0.252)
Telangana	0.146 (0.129 to 0.162)	0.137 (0.118 to 0.156)	0.252 (0.234 to 0.269)
Tripura	0.143 (0.113 to 0.175)	0.096 (0.067 to 0.123)	0.179 (0.151 to 0.210)
Uttar Pradesh	0.121 (0.111 to 0.131)	0.198 (0.188 to 0.209)	0.248 (0.238 to 0.257)
Uttarakhand	0.128 (0.106 to 0.150)	0.159 (0.137 to 0.183)	0.316 (0.293 to 0.338)
West Bengal	0.140 (0.130 to 0.151)	0.126 (0.114 to 0.138)	0.289 (0.276 to 0.301)

Abbr.: BMI = Body Mass Index, CI = Confidence interval.

Supplementary Table 19: Intracluster correlation coefficients for BMI, BG and BP as continuous variables at the community level by state

State	Intracluster Correlation Coefficient		
	Blood glucose (95% CI)	*Blood pressure (95% CI)*	*BMI (95% CI)*
Andaman and Nicobar Islands	0.023 (0.010 to 0.036)	0.093 (0.066 to 0.120)	0.078 (0.054 to 0.102)
Andhra Pradesh	0.046 (0.039 to 0.053)	0.034 (0.028 to 0.039)	0.110 (0.098 to 0.121)
Arunachal Pradesh	0.075 (0.065 to 0.083)	0.065 (0.056 to 0.075)	0.097 (0.086 to 0.109)
Assam	0.054 (0.044 to 0.063)	0.056 (0.046 to 0.067)	0.180 (0.156 to 0.207)
Bihar	0.138 (0.120 to 0.156)	0.078 (0.068 to 0.091)	0.114 (0.100 to 0.133)
Chandigarh	0.034 (0.016 to 0.053)	0.036 (0.018 to 0.057)	0.084 (0.052 to 0.119)
Chhattisgarh	0.062 (0.049 to 0.077)	0.063 (0.051 to 0.075)	0.117 (0.095 to 0.142)
Daman and Diu	0.079 (0.044 to 0.127)	0.064 (0.034 to 0.102)	0.084 (0.041 to 0.129)
Delhi	0.024 (0.018 to 0.030)	0.049 (0.039 to 0.060)	0.078 (0.065 to 0.091)
Goa	0.053 (0.032 to 0.075)	0.086 (0.063 to 0.115)	0.041 (0.024 to 0.059)
Haryana	0.049 (0.044 to 0.055)	0.047 (0.042 to 0.053)	0.089 (0.080 to 0.097)
Himachal Pradesh	0.042 (0.032 to 0.051)	0.058 (0.047 to 0.070)	0.096 (0.081 to 0.110)
Jharkhand	0.047 (0.038 to 0.060)	0.055 (0.043 to 0.068)	0.126 (0.103 to 0.151)
Karnataka	0.078 (0.073 to 0.084)	0.027 (0.025 to 0.031)	0.131 (0.123 to 0.139)
Kerala	0.057 (0.048 to 0.066)	0.168 (0.151 to 0.186)	0.129 (0.112 to 0.142)
Madhya Pradesh	0.094 (0.082 to 0.105)	0.100 (0.088 to 0.112)	0.114 (0.100 to 0.128)
Maharashtra	0.054 (0.049 to 0.059)	0.040 (0.036 to 0.043)	0.117 (0.110 to 0.125)
Manipur	0.065 (0.054 to 0.076)	0.045 (0.036 to 0.054)	0.074 (0.062 to 0.089)
Meghalaya	0.062 (0.049 to 0.076)	0.085 (0.069 to 0.104)	0.079 (0.062 to 0.096)
Mizoram	0.091 (0.077 to 0.107)	0.040 (0.032 to 0.048)	0.076 (0.064 to 0.088)
Nagaland	0.204 (0.179 to 0.229)	0.090 (0.073 to 0.105)	0.088 (0.071 to 0.103)
Odisha	0.043 (0.036 to 0.051)	0.083 (0.072 to 0.095)	0.149 (0.129 to 0.169)
Puducherry	0.024 (0.017 to 0.033)	0.019 (0.011 to 0.027)	0.059 (0.045 to 0.073)
Punjab	0.019 (0.016 to 0.021)	0.036 (0.033 to 0.040)	0.058 (0.053 to 0.063)
Rajasthan	0.111 (0.096 to 0.127)	0.070 (0.060 to 0.080)	0.163 (0.142 to 0.183)
Sikkim	0.029 (0.019 to 0.040)	0.026 (0.017 to 0.036)	0.049 (0.036 to 0.063)
Tamil Nadu	0.032 (0.029 to 0.036)	0.035 (0.032 to 0.039)	0.088 (0.082 to 0.095)
Telangana	0.050 (0.040 to 0.059)	0.061 (0.051 to 0.072)	0.099 (0.085 to 0.113)
Tripura	0.054 (0.037 to 0.074)	0.036 (0.022 to 0.053)	0.042 (0.029 to 0.060)
Uttar Pradesh	0.055 (0.048 to 0.063)	0.114 (0.102 to 0.128)	0.153 (0.135 to 0.171)
Uttarakhand	0.094 (0.070 to 0.118)	0.142 (0.109 to 0.175)	0.200 (0.160 to 0.246)
West Bengal	0.066 (0.059 to 0.074)	0.070 (0.062 to 0.078)	0.148 (0.135 to 0.160)

Source data are provided as a Source Data file.
Abbr.: BMI=Body Mass Index, CI= Confidence Interval.

Supplementary Table 20: Cluster characteristics by state

State	Number of clusters			Mean cluster size (SD)			Median cluster size (IQR)		
	Household	*Community*	*District*	*Household*	*Community*	*District*	*Household*	*Community*	*District*
Andaman and Nicobar Islands	2215	106	3	2.93 (1.37)	44.01 (8.98)	1507.89 (217.80)	3 (2)	44 (12)	1362 (474)
Andhra Pradesh	14209	797	13	3.44 (1.57)	48.05 (16.03)	2547.26 (494.91)	3 (2)	48 (23)	2413 (561)
Arunachal Pradesh	14461	583	16	3.02 (1.46)	59.79 (29.19)	1990.23 (296.73)	3 (2)	56 (18)	2092 (515)
Assam	21933	287	23	3.07 (1.40)	237.25 (126.51)	2137.16 (689.92)	3 (2)	215 (181)	1869 (1066)
Bihar	30059	395	33	3.02 (1.47)	190.94 (91.71)	1729.65 (547.54)	3 (2)	186 (118)	1718 (848)
Chandigarh	1307	50	1	3.48 (1.75)	65.24 (8.97)	3202.00 (0.00)	3 (2)	65 (13)	3202 (0)
Chhattisgarh	14720	197	16	3.24 (1.50)	220.64 (93.14)	1970.16 (353.88)	3 (2)	221 (140)	2011 (526)
Daman and Diu	805	44	1	3.60 (1.83)	41.95 (12.63)	1657.00 (0.00)	3 (3)	41 (19)	1657 (0)
Delhi	7386	440	9	3.55 (1.70)	49.13 (18.77)	2125.77 (701.34)	3 (2)	50 (26)	2334 (883)
Goa	2084	100	2	3.48 (1.59)	50.98 (14.52)	2330.25 (253.46)	3 (2)	50 (17)	2557 (510)
Haryana	24495	1254	21	3.56 (1.69)	51.97 (16.55)	2901.68 (769.26)	3 (2)	52 (23)	2921 (1217)
Himachal Pradesh	7627	454	12	3.40 (1.50)	37.56 (14.22)	1236.98 (275.50)	3 (2)	37 (18)	1310 (324)
Jharkhand	14461	201	16	3.33 (1.61)	187.64 (86.94)	1646.04 (184.80)	3 (2)	187 (130)	1700 (242)
Karnataka	37894	1811	30	3.84 (1.96)	56.82 (14.81)	3195.24 (563.90)	3 (3)	57 (20)	3262 (946)
Kerala	11573	664	14	3.25 (1.40)	42.02 (12.96)	1757.39 (520.28)	3 (2)	43 (17)	1685 (527)
Madhya Pradesh	34790	543	45	3.16 (1.40)	186.66 (104.29)	1639.58 (346.05)	3 (2)	168 (114)	1626 (550)
Maharashtra	39897	2017	35	3.91 (1.88)	52.81 (15.41)	2817.84 (620.02)	4 (3)	54 (20)	2843 (1025)
Manipur	8626	343	9	3.29 (1.43)	60.68 (11.79)	2234.98 (252.57)	3 (2)	61 (16)	2215 (524)
Meghalaya	5816	259	7	3.33 (1.58)	46.84 (11.99)	1627.58 (214.91)	3 (2)	46 (17)	1544 (277)
Mizoram	8380	320	8	3.36 (1.55)	68.72 (11.24)	2686.99 (239.90)	3 (2)	69 (14)	2700 (200)
Nagaland	6502	376	11	2.84 (1.26)	43.07 (16.03)	1245.24 (353.90)	2 (1)	44 (21)	1410 (542)
Odisha	32679	375	30	3.18 (1.45)	236.22 (98.19)	2291.22 (433.83)	3 (2)	233 (142)	2283 (768)

Puducherry	4381	200	4	3.60 (1.67)	59.75 (13.72)	2789.91 (370.44)	3 (2)	59 (16)	2998 (116)
Punjab	31793	1250	20	3.96 (1.72)	71.10 (12.32)	4408.83 (659.16)	4 (2)	71 (15)	4690 (939)
Rajasthan	25382	388	32	3.48 (1.62)	186.36 (96.74)	1648.60 (423.50)	3 (2)	185 (135)	1601 (755)
Sikkim	3922	160	4	3.08 (1.55)	56.32 (8.71)	2198.76 (98.28)	3 (2)	57 (13)	2186 (212)
Tamil Nadu	38633	1771	32	3.29 (1.49)	55.65 (13.70)	2870.65 (517.63)	3 (2)	56 (16)	2815 (743)
Telangana	9112	513	10	3.52 (1.66)	47.84 (15.81)	2122.86 (479.19)	3 (2)	48 (22)	1995 (791)
Tripura	2491	140	4	3.35 (1.46)	58.38 (16.83)	1483.96 (148.76)	3 (2)	61 (19)	1509 (339)
Uttar Pradesh	30987	653	70	3.56 (1.76)	172.30 (104.04)	1190.77 (609.83)	3 (2)	156 (144)	996 (981)
Uttarakhand	6914	153	11	3.08 (1.46)	224.28 (198.16)	1365.95 (501.89)	3 (2)	169 (201)	1342 (728)
West Bengal	20155	997	19	3.04 (1.53)	47.41 (14.22)	2280.69 (421.84)	3 (2)	48 (19)	2310 (614)

Source data are provided as a Source Data file.
Abbr.: SD=standard deviation, IQR=interquartile range

Supplementary Figures

Supplementary Figure 1: Intracluster correlation coefficients in relation to CVD risk factor prevalence by state

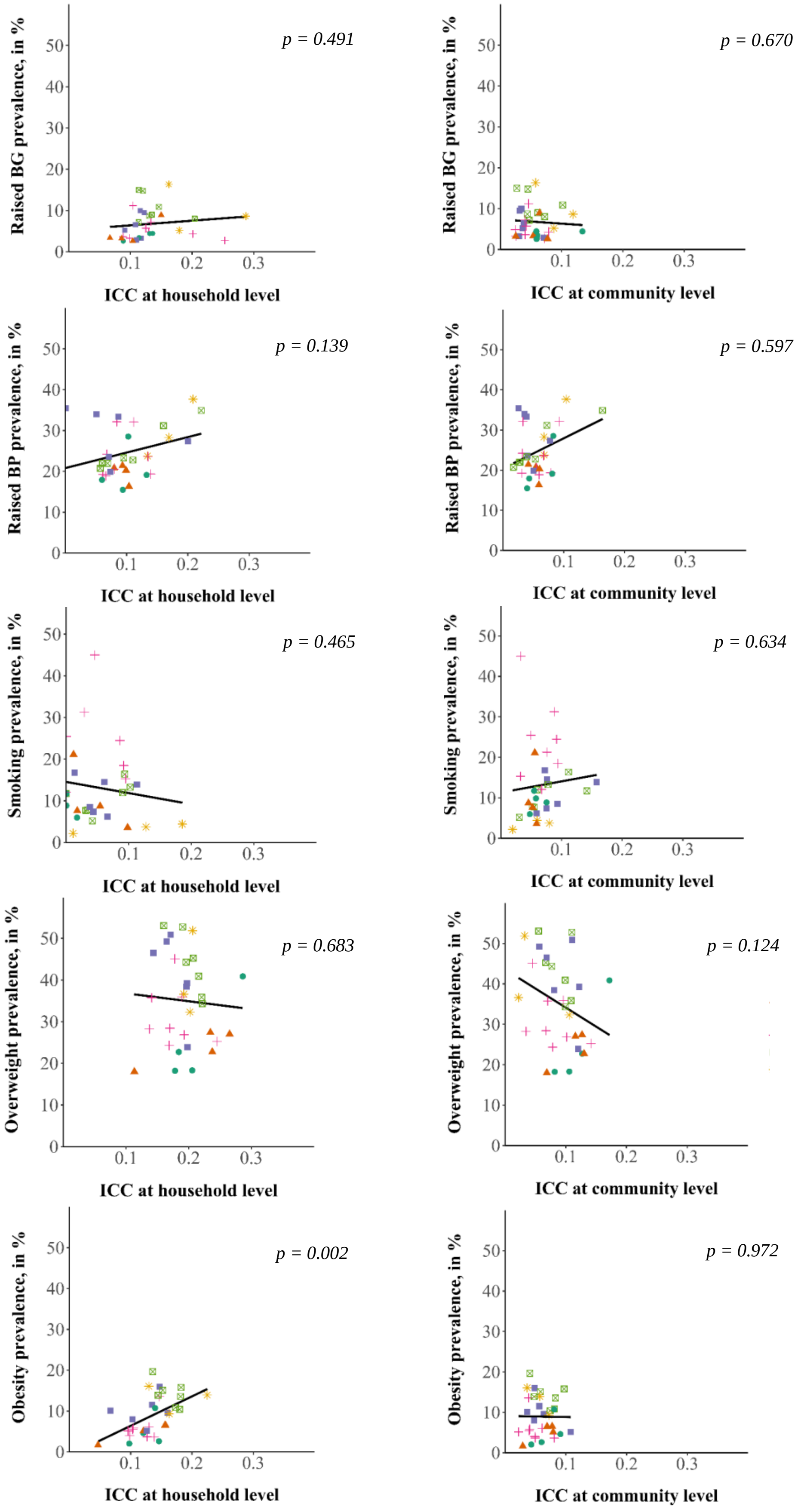

The black line is an ordinary least squares regression of the CVD risk factor prevalence per state onto the state-level ICC values (n=32). The p-value refers to the regression coefficient for this black line.
Each dot represents a state.
Colors designate the different zones in India as per the allocation of the Zonal Councils of the Government of India:green circle- central, orange triangle- east, purple square- north, pink cross- northeast, green square south, yellow star west. [1]
Source data are provided as a Source Data file.

Supplementary Figure 2: Intracluster correlation coefficients in relation to GDP per capita by state

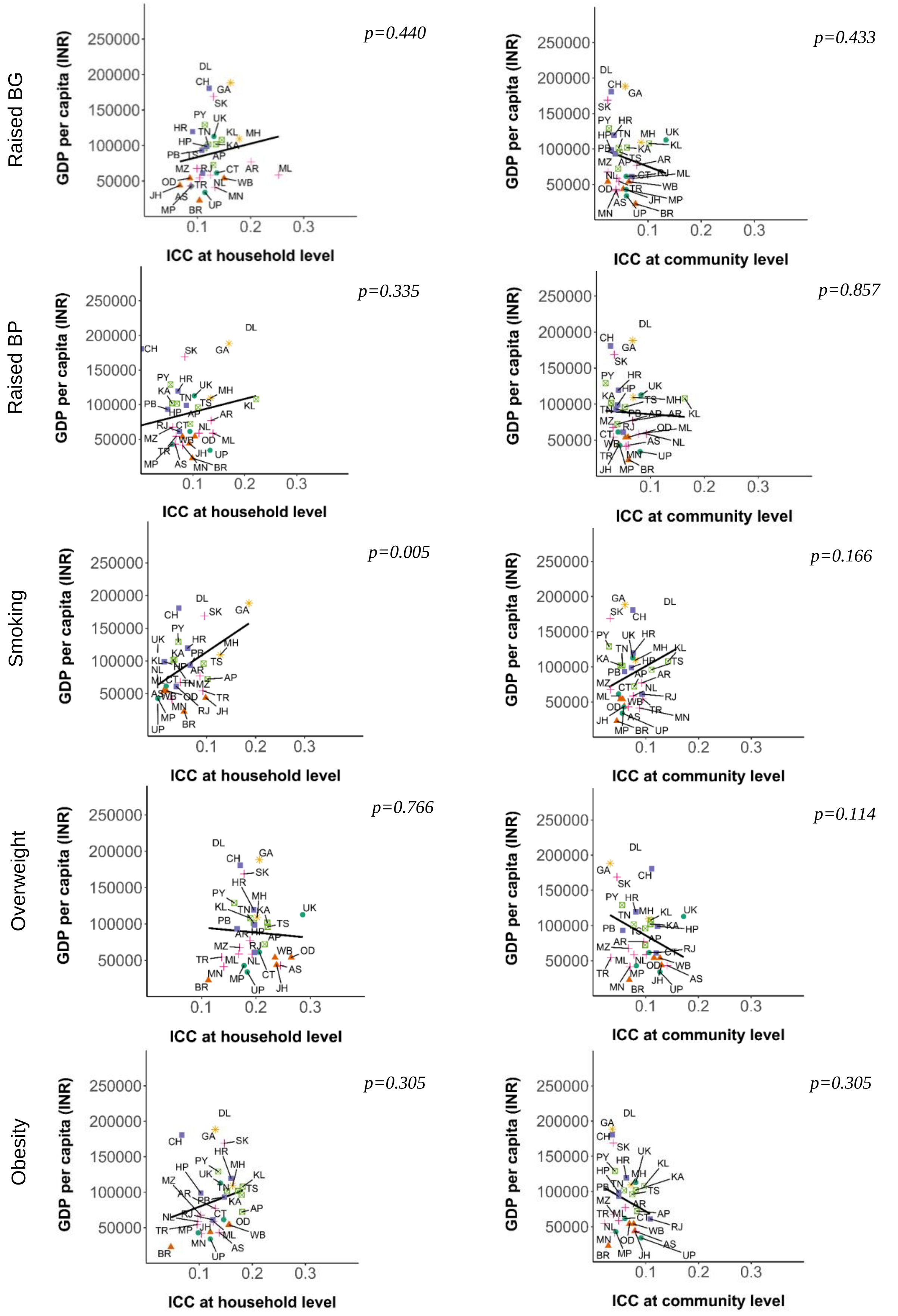

The black line is an ordinary least squares regression of the GDP per capita by state onto state-level ICC values (n=32). The p-value refers to the regression coefficient for this black line.
Abbr.: AN, Andaman and Nicobar Islands; AP, Andhra Pradesh; AR, Arunachal Pradesh; AS, Assam; BR, Bihar; CG, Chhattisgarh; CH, Chandigarh; DD, Daman and Diu; DL, Delhi;GA, Goa; HR, Haryana; HP, Himachal Pradesh; JH, Jharkhand; KA, Karnataka; KL, Kerala;MP, Madhya Pradesh; MH, Maharashtra; MN, Manipur; ML, Meghalaya; MZ, Mizoram; NL, Nagaland; OD, Odisha (Orissa); PB, Punjab; PY, Puducherry; RJ, Rajasthan; SK, Sikkim; TN, Tamil Nadu; TS, Telangana State; TR, Tripura; UP, Uttar Pradesh; UK, Uttarakhand (Uttaranchal); WB, West Bengal.
Colors designate the different zones in India as per the allocation of the Zonal Councils of the Government of India:green circle- central, orange triangle- east, purple square- north, pink cross- northeast, green square south, yellow star west. [1]
Source data are provided as a Source Data file.

Supplementary Figure 3. Intracluster correlation coefficients for BMI, BG and BP as continuous variables at the household and community level by state

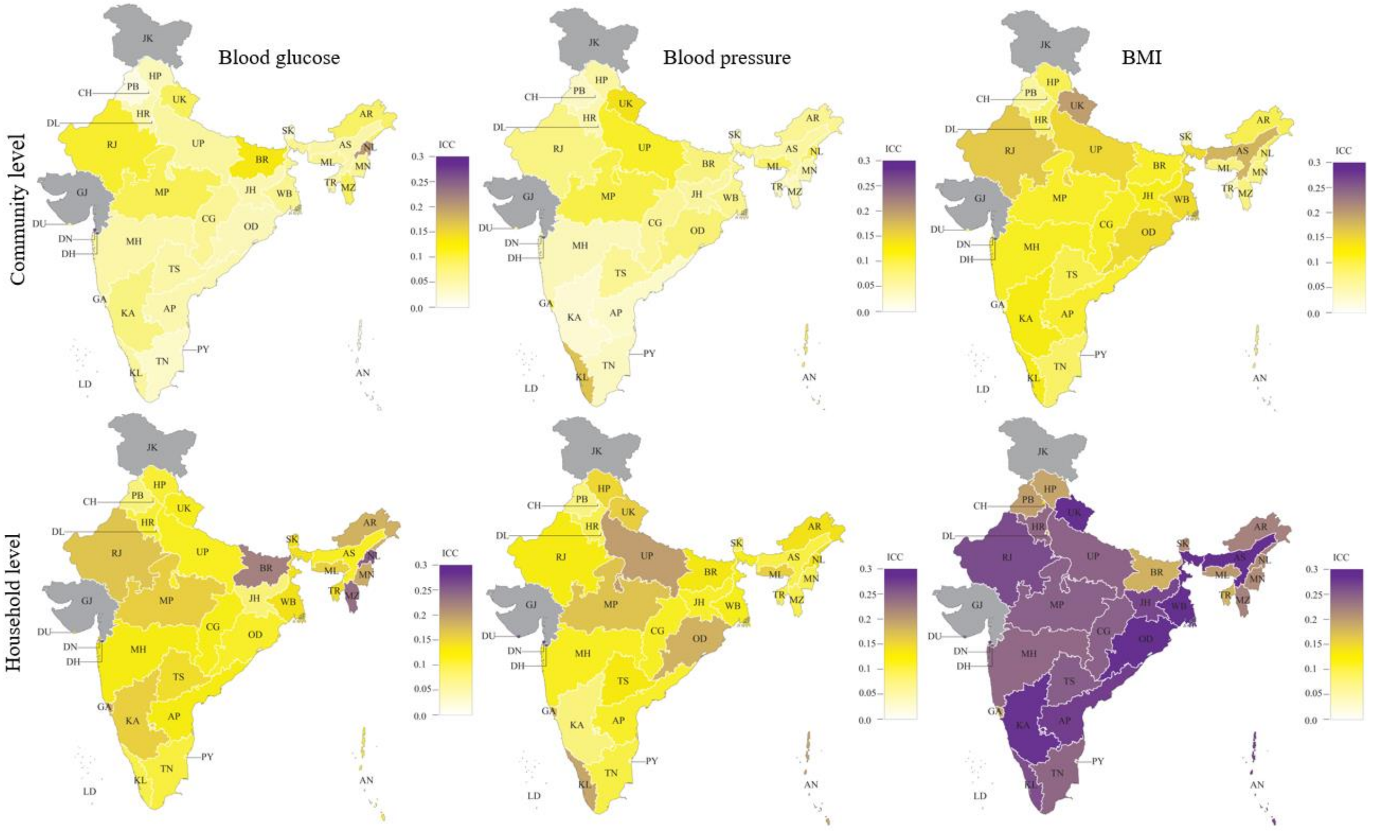

Abbr.: AN, Andaman and Nicobar Islands; AP, Andhra Pradesh; AR, Arunachal Pradesh; AS, Assam; BR, Bihar; CG, Chhattisgarh; CH, Chandigarh; DL, Delhi; DN, Dadra and Nagar Haveli; GA, Goa; HR, Haryana; HP, Himachal Pradesh; JH, Jharkhand; KA, Karnataka; KL, Kerala; ; MP, Madhya Pradesh; MH, Maharashtra; MN, Manipur; ML, Meghalaya; MZ, Mizoram; NL, Nagaland; OD, Odisha (Orissa); PB, Punjab; PY, Puducherry; RJ, Rajasthan; SK, Sikkim; TN, Tamil Nadu; TS, Telangana State; TR, Tripura; UP, Uttar Pradesh; UK, Uttarakhand (Uttaranchal); WB, West Bengal.
Source data are provided as a Source Data file.

Supplementary Figure 4: Intracluster correlation coefficients for BMI, BG and BP as continuous variables in relation to household wealth index by district, stratified by residency

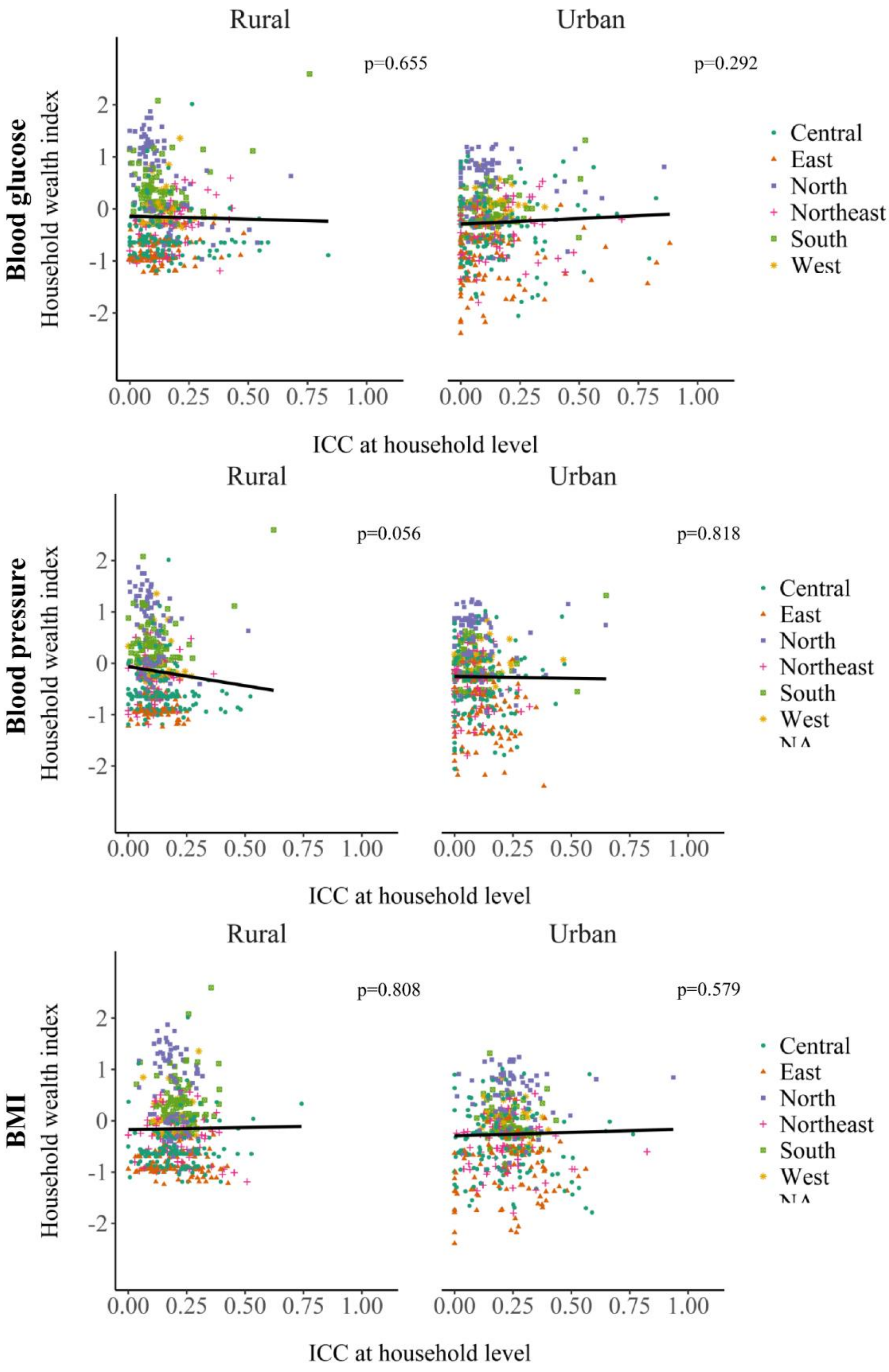

The black line is an ordinary least squares regression of district-level household wealth index onto household-level ICC with each district having the same weight. The p-value refers to the regression coefficient for this black line.
Colors designate the different zones in India as per the allocation of the Zonal Councils of the Government of India:green circle- central, orange triangle- east, purple square- north, pink cross- northeast, green square south, yellow star west. [1]
For the calculation of the ICCs we included districts with ≥ 50 participants and when separated by residency only districts with ≥ 20 participants. All 561 districts except for one had ≥ 50 participants. Then, for rural areas, 9 districts had ≤ 20 participants. For urban areas, 16 districts had ≤ 20 participants.
Source data are provided as a Source Data file.

Supplementary References

1. Ministry of Home Affairs. Zonal Council. New Delhi: Government of India; 2017. https://mha.gov.in/zonal-council
