# Supplementary material for: A cross-sectional study of cardiovascular disease risk clustering at different socio-geographic levels in India
Source: Nat Commun. 2020 Nov 18;11:5891. doi: 10.1038/s41467-020-19647-3 (PMC7674456; doi:10.1038/s41467-020-19647-3)
Supplement: Supplementary file 3 — Reporting Summary [file 41467_2020_19647_MOESM3_ESM.pdf]

nature research

Corresponding author(s): A.Bischops

Last updated by author(s): Oct 8, 2020

# Reporting Summary

Nature Research wishes to improve the reproducibility of the work that we publish. This form provides structure for consistency and transparency in reporting. For further information on Nature Research policies, see Authors & Referees and the Editorial Policy Checklist .

## Statistics

For all statistical analyses, confirm that the following items are present in the figure legend, table legend, main text, or Methods section.

| n/a | Confirmed | |
|---|---|---|
| ☐ | ☒ | The exact sample size (*n*) for each experimental group/condition, given as a discrete number and unit of measurement |
| ☐ | ☒ | A statement on whether measurements were taken from distinct samples or whether the same sample was measured repeatedly |
| ☐ | ☒ | The statistical test(s) used AND whether they are one- or two-sided<br>*Only common tests should be described solely by name; describe more complex techniques in the Methods section.* |
| ☐ | ☒ | A description of all covariates tested |
| ☐ | ☒ | A description of any assumptions or corrections, such as tests of normality and adjustment for multiple comparisons |
| ☐ | ☒ | A full description of the statistical parameters including central tendency (e.g. means) or other basic estimates (e.g. regression coefficient) AND variation (e.g. standard deviation) or associated estimates of uncertainty (e.g. confidence intervals) |
| ☐ | ☒ | For null hypothesis testing, the test statistic (e.g. *F*, *t*, *r*) with confidence intervals, effect sizes, degrees of freedom and *P* value noted<br>*Give P values as exact values whenever suitable.* |
| ☒ | ☐ | For Bayesian analysis, information on the choice of priors and Markov chain Monte Carlo settings |
| ☒ | ☐ | For hierarchical and complex designs, identification of the appropriate level for tests and full reporting of outcomes |
| ☐ | ☒ | Estimates of effect sizes (e.g. Cohen's *d*, Pearson's *r*), indicating how they were calculated |

*Our web collection on statistics for biologists contains articles on many of the points above.*

## Software and code

Policy information about availability of computer code

| | |
|---|---|
| Data collection | To collect the data of this study custom code written in R version 3.4.3 has been used which has been made publicly available in the "Harvard Dataverse" repository at https://doi.org/10.7910/DVN/NLU7HI. |
| Data analysis | Statistical analyses were performed in R version 3.4.3. All data cleaning and analysis code has been posted in the publicly available "Harvard Dataverse" repository at https://doi.org/10.7910/DVN/NLU7HI. |

For manuscripts utilizing custom algorithms or software that are central to the research but not yet described in published literature, software must be made available to editors/reviewers. We strongly encourage code deposition in a community repository (e.g. GitHub). See the Nature Research guidelines for submitting code & software for further information.

## Data

Policy information about availability of data

All manuscripts must include a data availability statement. This statement should provide the following information, where applicable:

- Accession codes, unique identifiers, or web links for publicly available datasets
- A list of figures that have associated raw data
- A description of any restrictions on data availability

Data is based on two publicly available datasets, which can be downloaded from https://nrhm-mis.nic.in/hmisreports/AHSReports.aspx, and http://www.iipsindia.ac.in (Annual Health Survey and District Level Household Survey Round 4 in India, specific AHS data names: CAB updated, COMB updated, MORT updated, WOMAN updated ; specific DLHS-4 data names: DLHS-4 cab, DLHS-4 HH, DLHS-4 EW, adultpopulationbystate).Furthermore, the cleaned datasets have been made available in the Harvard Dataverse repository at https://doi.org/10.7910/DVN/NLU7HI. All tables and figures are based on this dataset, the specific data used can additionally be found in a Source Data file.

# Field-specific reporting

Please select the one below that is the best fit for your research. If you are not sure, read the appropriate sections before making your selection.

- [ ] Life sciences
- [x] Behavioural & social sciences
- [ ] Ecological, evolutionary & environmental sciences

For a reference copy of the document with all sections, see nature.com/documents/nr-reporting-summary-flat.pdf

# Behavioural & social sciences study design

All studies must disclose on these points even when the disclosure is negative.

| | |
|---|---|
| Study description | Quantitative cross-sectional study with two population-based household surveys in India.The AHS was carried out from 2012 to 2013 and covered nine states (Assam, Bihar, Chhattisgarh, Jharkhand, Madhya Pradesh, Odisha, Rajasthan, Uttar Pradesh, and Uttarakhand), which were chosen because they had the highest rate of child and infant mortality in 2010. The DLHS-4 was carried out between 2012 and 2014, and covered all remaining states (except Gujarat for which data was not available in the public domain, and Jammu and Kashmir for which data was not collected due to violent conflicts) and five of seven Union Territories (UTs) (all except Dadra and Nagar Haveli, and Lakshadweep). Both surveys sampled non-pregnant adults aged 18 years and older. We pooled both datasets to receive a nationally representative sample in order to identify the variation of the level of clustering of CVD risk factors between risk factors and type of socio-geographic level (households, communities, districts, or states). |
| Research sample | Nationally representative sample of two population-based household surveys in India: the fourth District-Level-Household Survey (DLHS-4) and the second update of the Annual Health Survey (AHS). Jointly, these contain data on 1,618,359 participants aged at least 18 years. The rationale behind choosing this pooled dataset was to gain a nationally representative sample of Indian adults. |
| Sampling strategy | Two-stage cluster random sampling, stratified by rural-urban location (primary sampling units: villages in rural areas and census enumeration blocks (AHS) or urban frame survey blocks (DLHS-4) in urban areas; secondary sampling units: households, which were selected via systematic random sampling). |
| Data collection | First, the household head completed a questionnaire on sociodemographic information of all household members regardless of their presence at the interviewer's visit. Then all non-pregnant adult household members (aged ≥18) , which were present at the interviewer's visit, received height, weight, blood glucose (BG), and blood pressure (BP) measurements. Households were not revisited when eligible adults were not present for the interviewer's visit. Both the AHS and DLHS-4 measured BG in a capillary blood sample with a hand-held blood glucose meter (SD CodeFree [SD Biosensor, Gyeonggi, Republic of Korea]). The glucose meter converted capillary blood measurements to a plasma-equivalent value by multiplying the reading by 1.11. In both surveys, BP was measured twice in the left upper arm with a minimum of three minutes in between measurements using the Rossmax AW150 (Rossmax Swiss GmbH, Bernick, Switzerland). Weight was measured in both surveys with a digital scale, and height using a wall-mounted statute meter. Current tobacco smoking status was ascertained through self-report in the questionnaires. The researcher was not blinded to the test results or the questionnaire's answers. |
| Timing | 2012-2014 |
| Data exclusions | Out of 1,618,359 adults in the dataset, 1,103,476 (68.1%) participants had non-missing values for all cardiovascular disease risk factors (BG, BP, smoking status, height and weight) and were included in the analysis. Before conducting the analysis, we decided to exclude participants with missing values for at least one of the five CVD risk factors. 31.9% of participants were excluded. |
| Non-participation | The AHS and DLHS-4 did not publish response rates. |
| Randomization | Participants were not allocated to experimental groups. |

# Reporting for specific materials, systems and methods

We require information from authors about some types of materials, experimental systems and methods used in many studies. Here, indicate whether each material, system or method listed is relevant to your study. If you are not sure if a list item applies to your research, read the appropriate section before selecting a response.

## Materials & experimental systems

| n/a | Involved in the study | |
|---|---|---|
| x | | Antibodies |
| x | | Eukaryotic cell lines |
| x | | Palaeontology |
| x | | Animals and other organisms |
| | x | Human research participants |
| x | | Clinical data |

## Methods

| n/a | Involved in the study | |
|---|---|---|
| x | | ChIP-seq |
| x | | Flow cytometry |
| x | | MRI-based neuroimaging |

## Human research participants

Policy information about studies involving human research participants

| | |
|---|---|
| Population characteristics | The analysis included 1,103,476 adult participants . The DLHS-4 and AHS jointly contained data on 515,689 households, 17,841 communities, and 561 districts. 52.5% of participants were female and 42.0% were younger than 36 years. 9.1% of participants were obese (BMI≥27.5kg/m2), 7.7% had a raised blood glucose, and 26.9% had a raised blood pressure Smoking was far more common among men (23.3%) than women (2.3%). 39.0% of participants had received no formal schooling, 32.6% lived in an urban area, and 76.2% were currently married. |
| Recruitment | The DLHS-4 and AHS surveys employed two-stage cluster random sampling, stratified by rural-urban location. The primary sampling units (PSUs) – villages in rural areas and census enumeration blocks (AHS) or urban frame survey blocks (DLHS-4) in urban areas – were selected with probability proportional to population size. The secondary sampling units were households, which were selected via systematic random sampling (ie, sampling the first household randomly and then selecting every xth household). The household head completed a questionnaire on sociodemographic information of all household members (regardless of their presence at the interviewer's visit) and all non-pregnant adult household members (aged ≥18) received height, weight, blood glucose (BG), and blood pressure (BP) measurements. |
| Ethics oversight | The study received a determination of "Not Human Subjects Research" by the institutional review board of the Harvard T.H. Chan School of Public Health on 9 May 2018. |

Note that full information on the approval of the study protocol must also be provided in the manuscript.
